# Bibliotheca Medico-Chirurgica

**Published:** 1891-09

**Authors:** 


*Bibliotheca Medico-Chirurgica.* Göttingen: VANDENHOECK & RUPRECHT.

This bibliography, which has been recently added to our Exchange-list, deserves special notice. While not so voluminous as the *Index Medicus*, and in no sense a substitute for it, it is a most valuable work arranged in an admirable manner. Classified under a sufficient number of headings, the names of books are first given, and then, in different type, the titles of papers in the 80 journals which are laid under contribution. The enterprising compilers, who are to be warmly congratulated on their work, might well add to this number.

With much consideration for our linguistic deficiencies, they have provided us with a separate title and explanations in English; and we might take this, for which we thank them, as one more indication that our language is destined to be the universal one. The cheapness of this publication, for which Messrs. Luzac and Co. are the London agents, is a strong point in its favour, and we hope its circulation in this country will be largely increased; for although it is some years old, it does not seem to be as well known as it ought to be. The work is issued quarterly.

